# Precise diagnosis of pediatric posterior cranial fossa neoplasms based on 2.5D MRI deep learning

**DOI:** 10.3389/fonc.2025.1700694

**Published:** 2025-11-12

**Authors:** Jie Dong, Can Zhang, Yong Zhang, Limeng Zhao, Guohua Zhao, Wenjing Li, Yangyang Cheng, Xinxin Wang, Tan Ping, Xinyu Wang, Fupeng Wei, Qian Zhang, Yongqiang Li, Weijian Wang

**Affiliations:** 1 School of Information Engineering, North China University of Water Resources and Electric Power, Zhengzhou, China; 2 Department of Magnetic Resonance Imaging, The First Affiliated Hospital of Zhengzhou University, Zhengzhou, China; 3 School of Artificial Intelligence, Zhongyuan University of Technology, Zhengzhou, China

**Keywords:** pediatric posterior fossa tumor, 2.5D MRI, deep learning, cross-attention, interpretable AI

## Abstract

**Background:**

The MRI imaging traits of pediatric posterior cranial fossa neoplasms overlap highly, leading to difficulties in preoperative diagnosis. Their treatment strategies differ significantly, and traditional deep learning models have limitations in multi - sequence MRI fusion and clinical interpretability, so new solutions are urgently needed.

**Objectives:**

This study aims to develop a 2.5D multi - sequence MRI deep learning framework (ResSwinT) that integrates Residual Network and Swin Transformer, to achieve automatic classification of three main Pediatric posterior fossa tumors—Pilocytic astrocytoma (PA), Medulloblastoma (MB), and Ependymoma (EP), and enhance the interpretability of the model through the SHAP method, so as to provide a more reliable auxiliary decision-making basis for clinical practice.

**Methods:**

This study retrospectively collected 309 pediatric patients confirmed by pathology, including 109 PA, 130 MB and 70 EP. The MRI data of these patients included five sequences: T1WI, T1C, T2WI,FLAIR, and ADC. After preprocessing steps such as N4 bias field correction, resampling, sequence registration, and intensity normalization, samples were constructed using a 2.5D image construction strategy, and the ResSwinT model is designed. Its performance was compared with seven deep learning models such as Residual Network 18 and VGG16, and SHAP analysis was used to analyze trait contributions.

**Results:**

The proposed ResSwinT model outperforms existing commonly used deep learning models in all classification tasks, particularly showing outstanding performance in terms of area under the curve(AUC) and overall accuracy(ACC). For the PA vs Non-PA task: ACC 89.5%, AUC 0.975; for the MB vs Non-MB task: ACC 93.7%, AUC 0.978; for the EP vs Non-EP task: Acc 87.5%, AUC 0.937. SHapley Additive exPlanations(SHAP) analysis shows that the model pays high attention to the gross tumor volume and its surrounding structures, and its decision-making basis is highly consistent with key imaging biomarkers, verifying the interpretability and clinical relevance of the model.

**Conclusions:**

ResSwinT achieves high-precision classification of pediatric posterior fossa tumor through 2.5D multi-sequence fusion and cross-attention mechanism. SHAP attribution analysis reveals the biological basis of the model’s decision-making, providing clinicians with an interpretable AI-assisted diagnostic tool, and is expected to optimize individualized treatment strategies.

## 1 Introduction

Brain neoplasm is the most common solid tumor in children, with 45% to 60% occurring in the posterior cranial fossa (PF). Pilocytic astrocytoma (PA), Medulloblastoma (MB), and Ependymoma (EP) are the three most important types in the PF, and their diagnosis and treatment strategies are all different: PA only requires local resection, MB requires whole-brain radiation oncology, and EP surgery needs to avoid the brainstem (1, 2). However, the magnetic resonance imaging (MRI) traits overlap highly (e.g., both MB and EP are prone to calcium deposition and cystic degeneration), resulting in insufficient preoperative diagnostic accuracy. As the gold standard, pathological diagnosis has invasive risks, delays, and sample bias, and these limitations directly affect the formulation of surgical plans and patient prognosis (3, 4). Although traditional deep learning models have made progress in tumor classification, there are still significant limitations in multi-sequence MRI fusion and clinical interpretability.

Multi-sequence MRI reveals neoplasms traits from different dimensions. T1 weighted image (T1WI) can well display the anatomical structure of the brain, while T1 contrast + (T1C) shortens the relaxation time of T1WI by injection of gadolinium-based contrast media to better detect lesions with damaged blood-brain barrier. T2 weighted image (T2WI) is more sensitive to lesion information. Fluid-attenuated inversion recovery (FLAIR) sequence is easy to identify non-enhanced neoplasms and peritumoral oedema, while the ADC map obtained after processing the diffusion-weighted imaging sequence is a cellular marker and has been proven to be negatively correlated with cell proliferation. Therefore, these five MRI sequences all reveal different traits of human tissues from different angles and play a crucial role in early diagnosis and treatment (5–8).

In tumor analysis using multi-sequence MRI, precise tumor segmentation is a fundamental step for subsequent quantitative analysis. Its quality directly affects the reliability of feature extraction and ultimately determines the overall performance of the classification model. Currently, semi-automatic segmentation strategies have achieved good results in ensuring annotation quality. Meanwhile, emerging deep learning techniques offer new possibilities for more efficient segmentation annotation. Several studies (9, 10) have confirmed the feasibility of fully automatic segmentation in eliminating manual intervention. Other methods directly predict the entire image through an attention mechanism, thus avoiding an explicit segmentation process (11).

Although machine learning technology based on radiomics has shown clear clinical value in fields such as neurosurgical disease management and genetic gene analyses. However, previous machine learning models applied to the research of pediatric posterior cranial fossa neoplasms still have limitations in terms of accuracy and reproducibility; at the same time, existing deep learning methods also face challenges such as insufficient model interpretability (12–16). Although 2D Convolutional Neural Network (CNN) can extract local texture Traits, it loses the spatial continuity information of Neoplasms, resulting in insufficient recognition rate for Neoplasms with complex morphology. Although 3D models retain spatial information, they contain more noise, require processing massive voxel data, and have a cubic increase in computational complexity, making it difficult to deploy them in routine clinical practice (17).The 2.5D strategy proposed by Zhang (18) et al. improves classification accuracy while maintaining computational efficiency by introducing axial continuity information. Takao’s study showed that the 2.5D model utilizes continuity information between adjacent slices, effectively reducing the false positive rate, and the final model performance with a sensitivity of 88.7% and a positive predictive value of 58.9% is superior to the traditional 2D model, demonstrating the feasibility of applying 2.5D data in the field of deep learning (19, 20). Secondly, early Transformer models rely on global attention mechanisms and are insufficiently sensitive to key local details such as cystic changes in medical images. Swin Transformer optimizes long-range trait extraction through a shifted window mechanism, providing a new idea for solving the above problems. Although existing studies have attempted to integrate CNN with Transformer (e.g., ViT+Residual Network), there is a lack of local-global trait fusion mechanisms for medical images, and interpretability analysis has not been associated with multi-sequence MRI (21, 22). In addition, the accuracy of Ependymoma classification is low due to small sample size and trait confusion, so more robust trait extraction methods are urgently needed.

This study proposes a ResSwinT model based on 2.5D MRI, which realizes the mutual enhancement of local traits of Residual Network and global traits of Swin Transformer through cross-attention mechanism, and combines SHapley Additive exPlanations (SHAP) attribution analysis to reveal the anatomical basis for model decision-making, filling the technical gap between multi-sequence MRI fusion and clinical interpretability, and achieving automatic classification of 3 types of pediatric posterior cranial fossa brain neoplasms: Pilocytic astrocytoma, Medulloblastoma, and Ependymoma. This paper has the following contributions:

1. Propose a cross-attention module based on CNN and Transformer to realize dynamic fusion of local-global features.
2. Prove the role of 2.5D MRI sequences in classifying common types of neoplasms in the posterior cranial fossa of children.
3. Perform interpretability analysis on deep learning models to provide clinical decision-making basis.

## 2 Materials and methods

### 2.1 Data set

**Figure 1:**
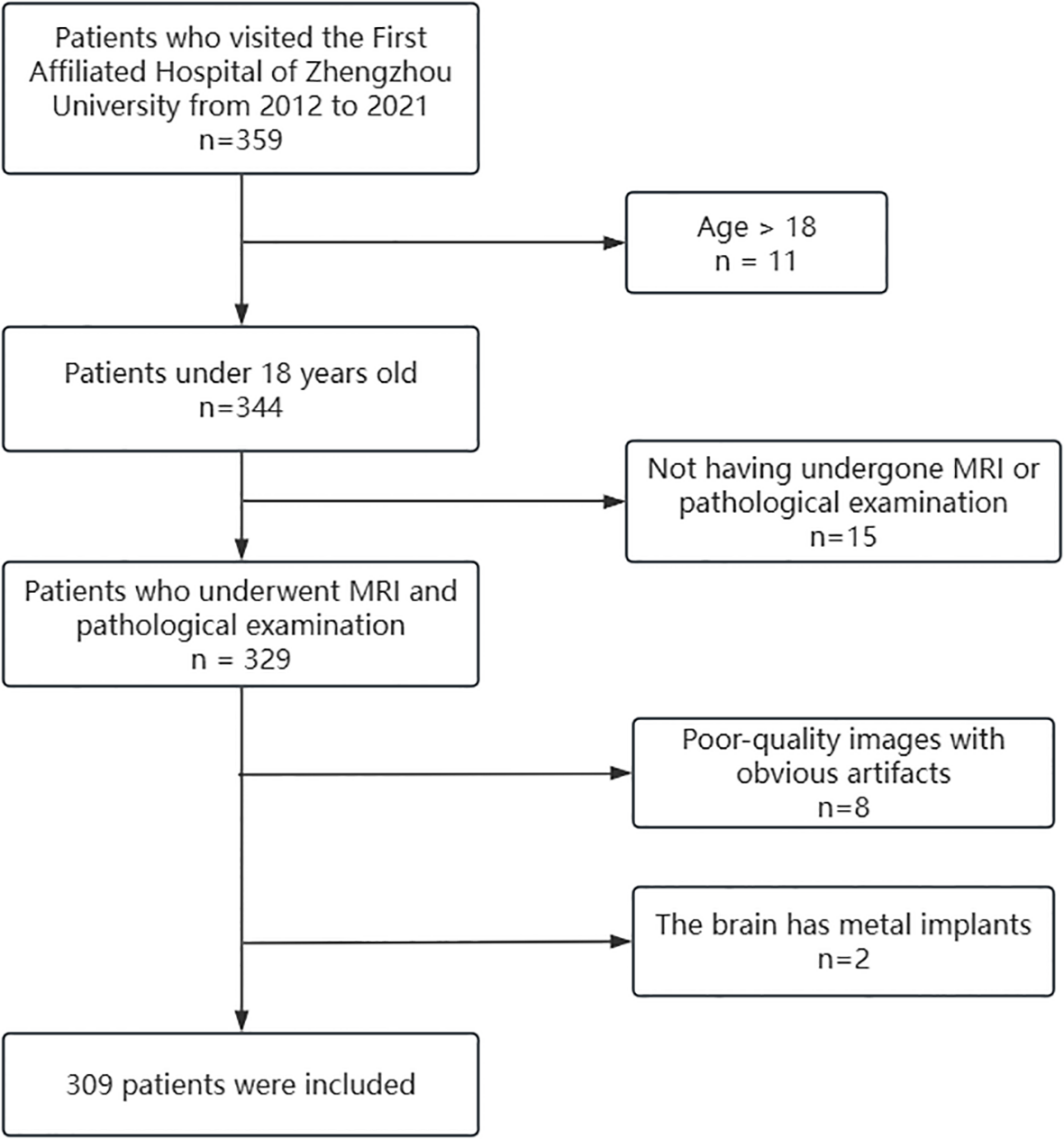
Diagram of the patient inclusion and exclusion process.

The dataset of this study was approved by the local ethics committee, and informed consent was waived. The patient selection process is shown in **Figure 1**. Inclusion criteria: 1. Pediatric patients aged less than 18 years; 2. Patients who completed preoperative MRI investigation and confirmed by pathology; 3. MRI sequences including T1WI, T1C, T2WI, FLAIR and ADC. Exclusion criteria: 1. Poor quality image with obvious artifacts. 2. Cerebrum containing metal implants. After screening, 309 cases were finally included in this study.

All imaging data were collected using a 3.0 T MRI scanner, and MRI imaging was performed using a standardized scan protocol. The field of view of the five acquired sequences (T1WI, T1C, T2WI, FLAIR, and ADC) was 220–240 mm, with a slice thickness of 6.5–7.0 mm; the in-plane resolution was concentrated at approximately 0.4–0.8 mm for T1/T1C/T2/FLAIR (approximately 0.6–1.45 mm for DWI). MRI scanners from different manufacturers achieved consistency in the above acquisition parameters, thus ensuring the comparability of imaging data across devices. The dataset was divided into training set, validation set, and test set in a ratio of 7:1.5:1.5.

### 2.2 Data preprocessing

To overcome the impact of different scanning devices on the robustness and reproducibility of image Traits, the preprocessing steps in this study include N4 bias field correction, resampling, multi-sequence registration, intensity normalization, etc. (23). First, N4 bias field correction is applied to the images to eliminate magnetic field inhomogeneity (3). Then, the T1C sequence is resampled to a resolution of 0.5 mm×0.5 mm×5 mm using 3D Slicer (version 5.6.2), and then the remaining sequences are registered to the T1C sequence using the General Registration (Elastix) method to ensure consistent spatial resolution across all MRI sequences. After that, intensity normalization is performed using the WhiteStripe Normalization processing method of CaPTK (version 1.9.0) to ensure consistent tissue contrast between different scanners (24, 25).

**Figure 2:**
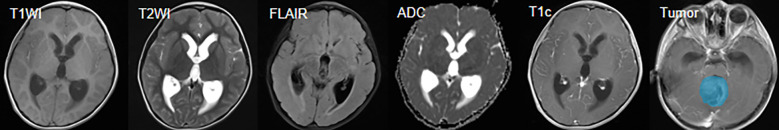
Image data and neoplasm mask.

In the gross tumor volume annotation phase, to ensure annotation quality while improving efficiency and controlling subjective differences, this study adopted a semi-automatic segmentation strategy: two radiologists with more than 5 years of experience independently delineated the gross tumor volume using semi-automatic segmentation functions in professional tools such as 3D Slicer, specifically including enhanced tumor (ET, referring to the part that shows enhancement in the T1C sequence compared to T1WI) and non-enhanced tumor (NET, referring to the part of the abnormal signal area within the tumor that does not belong to the enhanced or cystic component, such as the area showing abnormalities in T1WI/T2WI/FLAIR but not enhanced in T1C). All segmentation results were cross-reviewed and discussed for consensus to achieve annotation consistency and minimize the potential impact of inter-rater differences on model reproducibility (26). The multi-sequence MRI and the delineated gross tumor volume are shown in **Figure 2**.

### 2.3 Model construction

**Figure 3:**
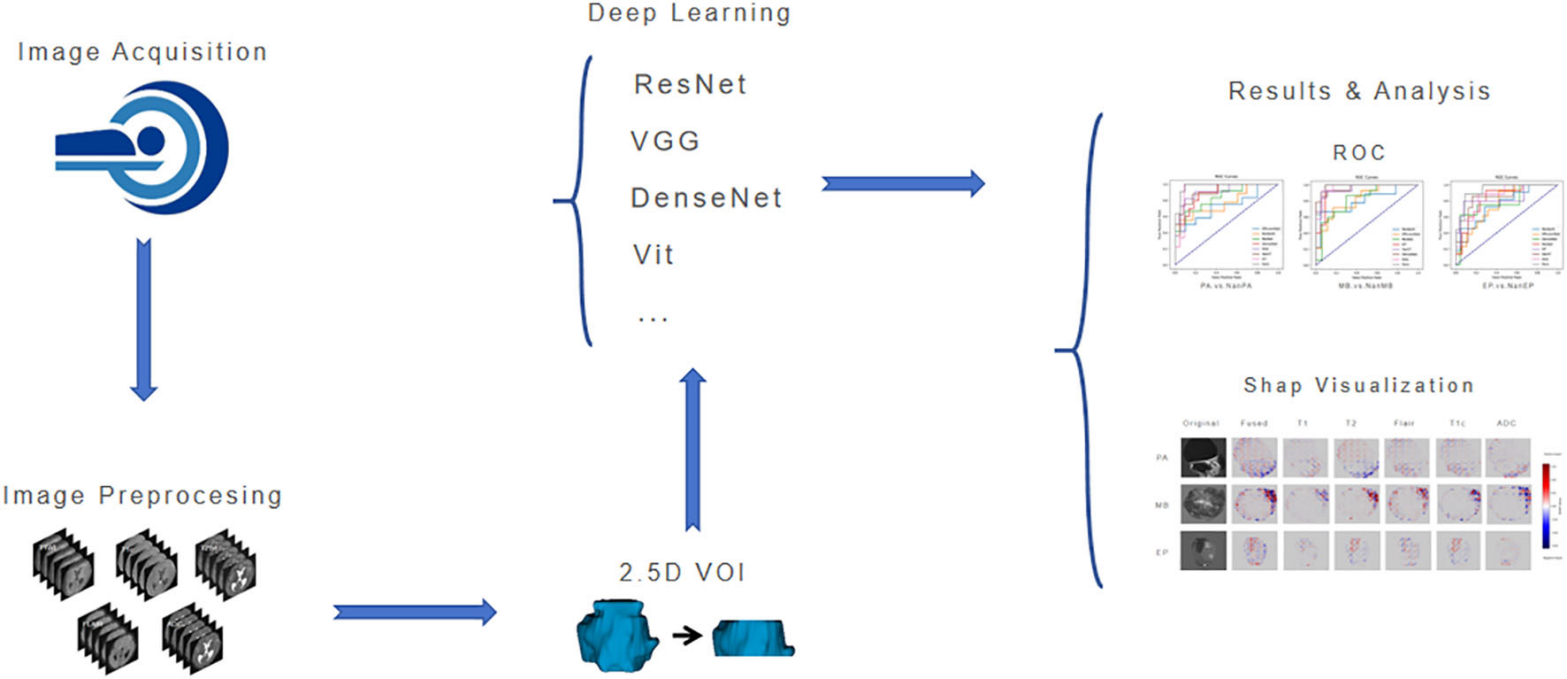
Workflow of this study.

The workflow of this study is shown in **Figure 3**. After image acquisition and preprocessing, model construction is performed. The model consists of a parallel dual-brace structure integrating Residual Network and Swin Transformer with 2.5D MRI, and the two branches are respectively used to extract local detail traits and global context traits in medical images. Then, comparison and evaluation of deep learning models are conducted, and finally SHAP visualization is used to increase the interpretability of model results.

#### 2.3.1 2.5D data construction strategy

To balance 3D spatial information and computational efficiency, this study constructed a 2.5D image dataset, that is, a total of five slices from the upper and lower parts of the maximum lesion slice in the data sample form a 2.5D data sample, and the final input is a 5-channel 2.5D image, which effectively retains the spatial continuity trait of the neoplasm. By using the improved ResSwinT as the deep learning model, five 2.5D MRI sequence data including T1WI, T1C, T2WI, FLAIR, and ADC were used as inputs to predict neoplasm categories.

#### 2.3.2 ResSwinT model architecture

**Figure 4:**
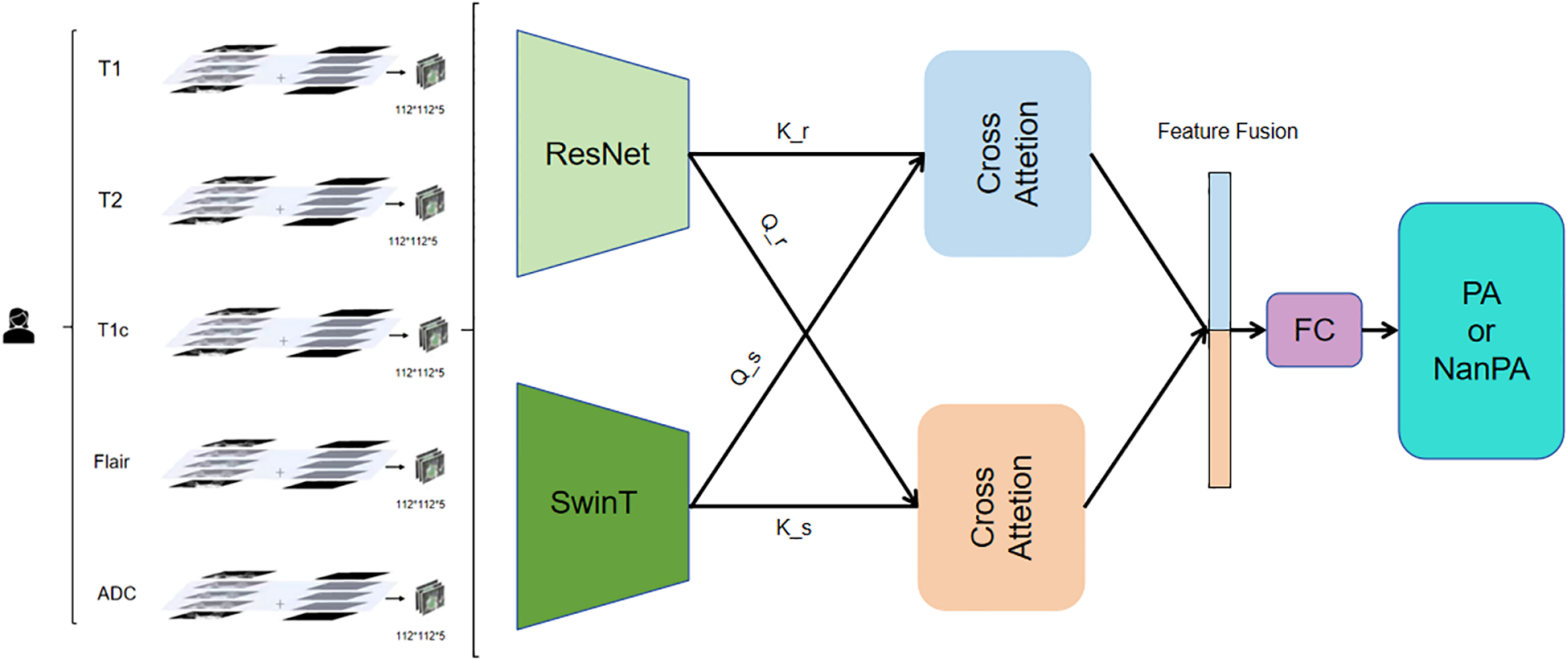
Model architecture diagram.

The model adopts a dual-branch parallel architecture (**Figure 4**), integrating the local feature extraction capability of CNN and the global modeling capability of Transformer. The Residual Network branch uses a residual network architecture to capture local Anatomy and topography traits in medical images. This branch first passes through a designed Stem layer, which uses 3*7*7 convolution kernels for moderate downsampling in the spatial dimension while maintaining high resolution in the depth dimension. The subsequent four-level residual structure adopts a progressive downsampling strategy, with each level containing two improved 3D residual blocks. The residual connection design ensures stable Transmission of gradients and solves the problem of deep network training. Moreover, it gradually transitions from basic features such as edges and textures to complex high-level features, laying the foundation for subsequent global feature fusion. The hierarchical structure and window-based self-attention mechanism of the Swin Transformer branch capture long-range dependence relationships between traits at different scales, deeply explore the value of global traits in medical image classification, and can especially capture potential connections between distant locations in different regions of multimodal images, thereby enhancing the model’s understanding of overall image traits. Six alternately stacked Swin Transformer blocks form the backbone, where regular window attention focuses on local region computation, and the shifted window mechanism achieves cross-window information interaction through three-dimensional cyclic displacement (27, 28).

To address the challenge of dual-branch feature alignment and fusion, this work employs a 2.5D cross-attention mechanism. This module establishes a bidirectional feature interaction channel: one direction utilizes the Residual Network features as queries and the Swin Transformer features as keys and values, thereby enriching local details with global contextual guidance; conversely, the other direction employs Swin Transformer features as queries and Residual Network features as keys and values, effectively injecting fine-grained local information into the global representation.

The fusion process comprises four key stages: First, feature map sizes are unified via three-dimensional adaptive pooling to resolve spatial resolution discrepancies. Next, bidirectional attention weight matrices are computed to capture complex inter-feature relationships. Then, heterogeneous features are fused through a shared value transformation layer. Finally, a learnable weighting coefficient γ is introduced to adaptively control the fusion intensity, allowing the model to dynamically balance the contributions from both branches.

Essentially, this module implements a mutual enhancement mechanism: Residual Network features gain discriminative power through the global semantics provided by the Swin branch, while Swin Transformer features acquire improved boundary perception via the local details from the Residual Network branch.

#### 2.3.3 Training configuration

This study adopted a standardized training protocol to ensure the consistency and reproducibility of the experimental process. All models were trained under a unified hyperparameter configuration, such as a batch size set to 16, a maximum number of training epochs set to 200, and an early stopping mechanism (with a patience value set to 10 training epochs). The learning rate scheduling strategy employed the cosine annealing with warm restarts algorithm, providing a consistent dynamic adjustment scheme of learning rate for all models.

To preserve the original traits of medical imaging data and their clinical relevance, any data augmentation techniques were strictly avoided during the training process. In view of the inherent characteristics of different model architectures, we achieved an optimal balance between learning efficiency and classification accuracy for each model through meticulous parameter refinement while maintaining the consistency of training conditions.

### 2.4 Performance evaluation

To verify the superiority of the ResSwinT model, this study designed comparative experiments. The comparative models include seven mainstream SOTA deep learning models (Residual Network18, VGG16, EfficientNetB0, ResNeXt, DenseNet121, Vit, Swin Transformer) and their performance on three binary classification tasks (PA vs NanPA, MB vs NanMB, EP vs NanEP). All models were evaluated under the conditions of the same test set and 2.5D multi-sequence input. The performance evaluation metrics mainly include the following: Area Under the Curve (AUC), which reflects the model’s ability to distinguish between positive and negative samples, obtained by plotting the ROC curve and calculating the area under the curve. Accuracy(ACC) measures the proportion of the model’s overall correct predictions, that is, the proportion of correctly predicted positive and negative samples to the total samples.F1-score is used as a comprehensive evaluation index to balance precision and recall, and has advantages for imbalanced data. Precision measures the proportion of samples predicted as positive by the model that are actually positive; high precision means the model is more reliable in predicting positive samples. Sensitivity(also known as recall) measures the model’s ability to find all positive samples; high recall means the model can effectively identify positive samples. Specificity measures the model’s ability to correctly identify negative samples.

### 2.5 Statistical analysis and interpretability

SPSS statistical software (version 31.0) was used for data analysis in this study. Continuous variables were described as Mean ± Standard Deviation, and analysis of variance was used for comparison between groups. Categorical variables (such as gender, neoplasm grade, neoplasm location) were described as Frequencies and Percentages, and the Chi-square test was used for comparison between groups. Statistical significance was defined as a two-tailed p-value < 0.05. SHAP values were used in interpretability analysis to quantify the contribution of each MRI sequence to the prediction, and fused SHAP plots were generated to comprehensively display the model’s attention regions, revealing the key role of neoplasm and its boundary structures in the decision-making process.

## 3 Results

### 3.1 Analysis of clinical features of patients

The traits of three groups of patients, including PA (n=109), MB (n=130), and EP (n=70), were compared (**Table 1**). According to the definition of the WHO classification (29), these tumor types have different inherent biological invasiveness: the PA cohort consists entirely of CNS WHO grade 1 tumors, the MB cohort consists of CNS WHO grade 4 tumors, and the EP cohort includes tumors classified by their type (CNS WHO grade 2 or 3). In addition to these defining features, statistical analysis revealed significant differences in patient age (p=0.006) and the most critical tumor location based on image diagnosis (p<0.001). The above statistical differences indicate that it is feasible to achieve automated differential diagnosis of these three types of tumors based on MRI images.

**Table 1:** Analysis of clinical characteristics of patients.

Variables	PA(n=109)	MB(n=130)	EP(n=70)	t/Z/χ² value	p-value
Age (mean ± standard deviation)	8.0 ± 4.2	8.8 ± 4.1	9.6 ± 5.0	5.21	0.006
Gender				1.91	0.384
Male	65	81	38		
Female	44	45	32		
Tumor grade				210.5	<0.001
Low-grade	109	0	12		
High-grade	0	130	58		
Tumor location				88.4	<0.001
Fourth ventricle	20	85	45		
Cerebellum	70	30	6		
Others	19	11	19		

### 3.2 Comparison of performance with other SOTA models

**Figure 5:**
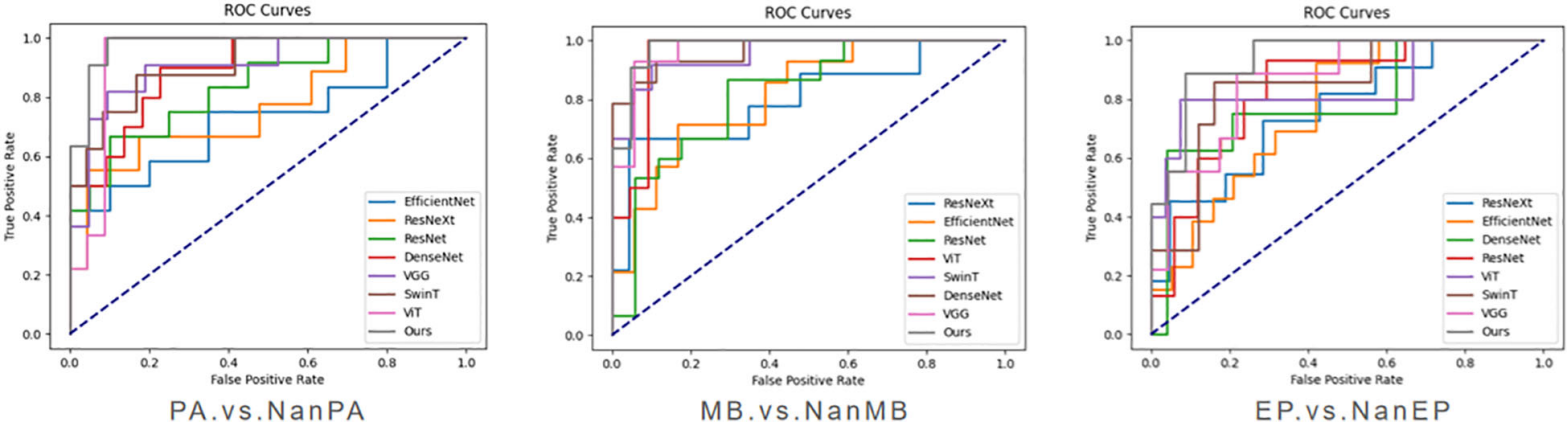
ROC comparison graphs of various models under different classification tasks.

To verify the effectiveness of the proposed model on our dataset, we conducted three groups of experiments (PA.vs.NanPA、MB.vs.NanMB、EP.vs.NanEP) to evaluate the performance of all methods. The ResSwinT model showed superior performance in all three classification tasks, and the results are shown in **Tables 2**–**4** and **Figure 5**.

**Table 2:** PA.vs.NanPA Performance comparison of various models under classification tasks.

Task	Model	Auc	Accuracy	Recall	Precision	F1	Specificity
PA. vs. NanPA	ResNet18	0.826	0.764	0.390	0.840	0.528	0.959
	VGG16	0.911	0.840	**0.878**	0.698	0.776	0.824
	EfficientNetB0	0.731	0.788	0.514	0.803	0.579	0.942
	ResNeXt	0.768	0.778	0.550	0.856	0.537	**0.966**
	DenseNet121	0.894	0.814	0.544	0.869	0.657	0.960
	Vit	0.934	0.873	0.759	0.840	0.791	0.934
	Swin	0.929	0.864	0.720	0.860	0.775	0.941
	Ours	**0.975**	**0.895**	0.801	**0.870**	**0.827**	0.942

Bold values means the highest value for each metric in the tables.

**Table 3:** MB.vs.NanMB Performance comparison of various models under classification tasks.

	Model	Auc	Accuracy	Recall	Precision	F1	Specificity
MB.vs. NanMB	ResNet18	0.851	0.725	0.501	0.843	0.623	0.921
	VGG16	0.968	0.895	0.900	0.872	0.888	0.887
	EfficientNetB0	0.765	0.726	0.524	0.679	0.595	0.804
	ResNeXt	0.802	0.777	0.500	0.848	0.540	0.910
	DenseNet121	0.960	0.892	0.858	**0.901**	0.876	0.923
	Vit	0.964	0.906	0.816	0.850	0.800	0.936
	Swin	0.954	0.913	**0.951**	0.862	**0.903**	0.884
	Ours	**0.978**	**0.937**	0.800	0.846	0.856	**0.939**

Bold values means the highest value for each metric in the tables.

**Table 4:** EP.vs.NanEP Performance comparison of various models under classification tasks.

	Model	Auc	Accuracy	Recall	Precision	F1	Specificity
EP.vs. NanEP	ResNet18	0.835	0.719	0.549	0.835	0.660	0.882
	VGG16	0.865	0.781	0.750	0.695	0.667	0.865
	EfficientNetB0	0.818	0756	0.550	0.679	0.595	0.803
	ResNeXt	0.762	0.750	0.418	**0.856**	0.539	0.889
	DenseNet121	0.791	0.769	0.515	0.844	0.656	0.833
	Vit	0.845	0.844	**0.761**	0.778	**0.737**	0.910
	Swin	0.846	0.813	0.600	0.667	0.400	0.941
	Ours	**0.937**	**0.875**	0.625	0.758	0.500	**0.976**

Bold values means the highest value for each metric in the tables.

As shown in **Tables 2**, **3**, and **4**, our model outperforms all commonly used models in terms of ACC and AUC in the three tasks, demonstrating its superior classification ability.

In the PA vs NanPA task, the ResSwinT model ranked first in accuracy (89.5%), AUC (97.5%), recall (80.1%), and F1-score (82.7%), while maintaining high specificity (94.2%), indicating that the ResSwinT model is more balanced in identifying positive and negative samples. In the MB vs NanMB task, although Swin Transformer performed the best in recall (95.1%) and F1-score (90.3%), the ResSwinT model outperformed Swin Transformer in accuracy (93.7% vs 91.3%), AUC (97.8% vs 95.4%), and specificity (93.9% vs 88.4%), reflecting better overall performance. In the EP vs NanEP task, due to the imbalance between recall and precision, the F1-score (50.0%) of the ResSwinT model was lower than that of models such as Vit (73.7%) and VGG16 (66.7%).However, in scenarios requiring high specificity (such as avoiding incorrect diagnosis of non-EP as EP), the ResSwinT model has significant advantages.

It is worth further pointing out that, to comprehensively assess mock-up efficiency, we compared the quantity of arguments and floating point operations of each mock-up. As shown in **Table 5**, although ResSwinT has a higher quantity of arguments (56.21M) than most CNN mock-ups, its computational complexity (94.99G FLOPs) is significantly lower than that of VGG16 (555.51G) and Swin Transformer (249.49G). This outcome indicates that the performance boosts achieved by ResSwinT do not stem from simple spread of argument quantity, but rather benefit from its efficient schema Design that fuses local and global traits.

**Table 5:** Complexity comparison of different models.

Model	ResNet18	VGG16	EfficientNetB0	ResNeXt	DenseNet121	Vit	Swin	Ours
Params(M)	33.19	63.06	4.07	14.95	11.27	3.24	27.49	56.21
FLOPs (G)	34.40	555.51	3.17	20.98	69.72	30.54	249.49	94.99

Experimental results show that the ResSwinT model performs excellently in all three classification tasks, and consistently outperforms all commonly used models especially in ACC and AUC. In the PA and MB tasks, the ResSwinT model is also competitive in indicators such as recall and F1 score; in the EP task, although the relatively low recall leads to a low F1 score, the extremely high specificity indicates that this model has unique advantages in reduction of false positives. Therefore, the proposed ResSwinT model has superior performance in the classification of pediatric posterior cranial fossa neoplasms.

### 3.3 SHAP analysis

**Figure 6:**
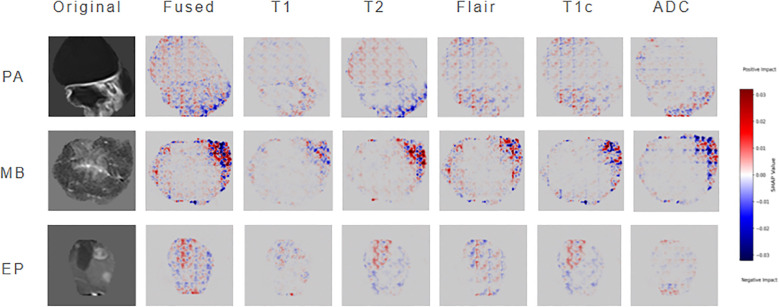
Shap analysis diagram.

To enhance model interpretability, this study employs SHAP to perform attribution analysis on ResSwinT’s prediction outcomes. In neuroimaging diagnostic practice, the lesion slice at the maximum cross-section typically contains the richest diagnostic information, including tumors’ characteristic internal structures, boundary features, and their anatomical relationships with surrounding critical structures. Based on this clinical rationale, the central slice data of the largest lesion plane was selected as input, mitigating the dilution effect of non-diagnostic regions—potentially introduced by equidistant slicing—on key features (30, 31).The specific results are shown in **Figure 6**, which presents three types of visualization images: original MRI images, fused SHAP hot maps, and individual SHAP hot maps of the five modalities. The original images are used to intuitively present the imaging traits of the neoplasm; the fused SHAP hot maps are generated by the method of maximum absolute value while retaining the sign, showing the regions that the model focuses on the most as a whole, where red regions indicate positive contributions to the classification result and blue regions indicate negative contributions; the modality-specific SHAP hot maps reveal the independent roles of different MRI sequences in classification discrimination.

In cases of PA, the SHAP hot map shows that the model has obvious attention to the cystic hyperintense regions of NET in T2 and FLAIR sequences, and this region presents strong highlighting in the fusion map. This is consistent with the common cystic degeneration trait of PA, suggesting that the model mainly relies on the signal characteristics of cystic and surrounding oedema when identifying PA. In MB cases, the fused SHAP heatmap focuses on the ET part of the neoplasm, where the low ADC regions of the ADC sequence are particularly prominent; meanwhile, the T2 sequence also emphasizes the signal of ET; at the same time, the T2 sequence also emphasizes the signal of CET. This indicates that the model mainly refers to the restricted diffusion regions caused by high cell density when distinguishing MB, and combines the solid manifestations on T2 for judgment, which is highly consistent with the pathological characteristics of MB. In EP cases, the model showed strong attention to the heterogeneous enhancement areas of ET in the T1C sequence, and the fused SHAP hot map also highlighted the enhancement areas and their distribution along the ependyma. In addition, some NET boundary areas of T2 and FLAIR were also marked by SHAP, suggesting that the model not only depends on the enhancement pattern of ET but also refers to the spatial relationship between NET and cerebral ventricle structure when identifying EP.

In conclusion, through the layer-by-layer analysis of original images, fused hot maps, and modality-specific hot maps, the model discrimination basis for different neoplasms can be clarified: PA mainly depends on the cystic signal and boundary trait of T2/FLAIR, MB mainly depends on the diffusion restriction trait of ADC/T2, and EP mainly depends on the heterogeneous enhancement manifestation of T1C. This result not only verifies that the discrimination logic of the model is highly consistent with clinical experience, but also reveals the multimodal discrimination mechanism of the model in easily confused cases, thereby significantly improving the interpretability and clinical application value of the model.

## 4 Discussion

Preoperative MRI diagnosis of pediatric posterior fossa tumor faces challenges due to overlapping imaging traits. Although deep neural networks serve as a solution, early studies are limited by sample size and the number of MRI sequences, resulting in limited classification accuracy and failure to fully unleash the technical potential. Subsequent CNNs (such as ResNeXt and EfficientNet) have achieved an accuracy exceeding 90% in a large sample set of 617 cases by virtue of their advantage in local trait extraction. While Transformers (such as ViT and Swin Transformer) optimize global trait modeling through self-attention mechanisms, problems such as difficulty in trait alignment, low data utilization efficiency, insufficient exploration of clinical value, and deletion of interpretability still exist when the two types of models are combined (32–35).

The ResSwinT model proposed in this study utilizes the advantages that CNN is good at extracting local anatomical traits and Transformer is strong at capturing global context, and the cross-attention module realizes alignment through bidirectional trait interaction: using Residual Network local traits as queries and Swin traits as keys and values to obtain global guidance, while using Swin global traits as queries and Residual Network traits as keys and values to inject local details. This mechanism improves the AUC of the MB vs NonMB task to 0.978, which is 2.4 percentage points higher than that of Swin Transformer, with an accuracy rate of 93.7%, verifying the gain of trait alignment on classification performance. For example, the hierarchical feature Fusion schema can be further explored, and interaction mechanisms can be embedded in multiple network layers, thereby achieving more robust and refined classification performance in complex scenes (36–39).

To address the limitation of low data utilization efficiency, this study proposes an improved 2.5D policy to balance spatial information retention and computational efficiency. Existing studies show that 2D models have limited performance in EP taxonomy due to the lack of axial information. For example, Zhou et al. achieved an AUC of only 0.84, and Quon et al.’s integration scheme achieved an AUC of 0.87 for differentiating MB from EP (40, 41). Although 3D models can completely retain spatial structures, the sharp increase in voxel volume leads to a significant elevation in computational complexity, limiting the feasibility of clinical application. The 2.5D scheme in this study achieves data dimensionality reduction while maintaining axial continuity, and achieves performance breakthrough by combining the cross-attention module. The experiment results show that in the EP vs Non-EP classification task, this policy can reach an AUC of 0.937, with inferencing efficiency approximately three times higher than that of 3D models (about 30 seconds per case) and a specificity of 97.6%.This design effectively mitigates the contradiction between spatial info loss and computational complexity, provides an efficient and high-quality data foundation for cross-attention trait fetching, and highlights the mock-up’s performance advantages in pediatric posterior fossa tumor taxonomy.

The ResSwinT model in this study balances the performance across the three major subgroups of pediatric posterior fossa tumor, overcoming the limitation of existing models where some subgroups perform well while others perform poorly. For example, the RF model by Novak et al. has an overall classification accuracy of 86.3%, but the recall rate for EP is only 62.5%; the model by Quon et al. has an overall accuracy as high as 92%, but the AUC for distinguishing EP from MB is only 0.87 (12, 41). In contrast, the accuracy of all subgroups in this study exceeds 87%, with an average AUC of 0.963. From the perspective of clinical application scenarios, this model can meet different diagnostic and treatment needs. For instance, the high accuracy for PA supports the determination of preoperative local resection plans, the AUC of up to 0.978 for MB facilitates the formulation of radiation oncology decisions, and the high specificity for EP can ensure Surgery safety to a certain extent, forming clinical diagnostic support for all subgroups. In addition to diagnostic efficacy, the computational efficiency of this model fully meets the requirements for immediate preoperative auxiliary diagnosis.

Meanwhile, the black-box trait of deep learning is a major obstacle to clinical implementation. This study constructs the association between the model and neoplasms pathological traits through SHAP analysis, making the diagnostic results traceable. In PA, the cystic hyperintense areas on T2/FLAIR are focused on; in MB, the diffusion-restricted areas reflecting high cell density on ADC/T2 sequences are emphasized; and in EP classification, the heterogeneous enhancement areas on T1C are highlighted. The results are highly consistent with clinical pathological cognition. For example, Dong et al.’s paper pointed out that ADC values are negatively correlated with neoplasms cell density (42). The high attention paid to ADC sequences by the model in this study further verifies this pathological-imaging association, enabling clinicians to assist in judgment through the model’s focused regions instead of simply relying on probability output, which significantly improves clinical trust. Therefore, the fusion and interpretation of multi-sequence magnetic resonance imaging not only verifies the value of multi-sequence data integration in practice, but also provides a feasible path for building a reliable image-assisted diagnosis tool (43).

Overall, the ResSwinT model based on 2.5D multi-sequence MRI proposed in this study provides a new solution for the accurate diagnosis of pediatric posterior cranial fossa neoplasms. Although this study has the limitation of a single-center retrospective design (44), which may affect the generalization ability of the model in a wider population, we have maximized the robustness of the model through methods such as including different models of scanners from six major mainstream manufacturers and adopting strict image pretreatment procedures in the study design. Importantly, this study has successfully achieved the effective integration of CNN and Transformer architectures in 2.5D multi-sequence MRI in this field, and verified the consistency between its decision-making mechanism and clinical cognition through interpretability analysis (45). These achievements not only provide an important methodological reference for AI-assisted diagnosis of pediatric posterior cranial fossa neoplasms but also lay a solid foundation for future multicenter validation studies. Promoting multicenter external validation and exploring the integration of radiomics and genomics will be the key directions of our subsequent work (13, 37).

## 5 Conclusion

This study proposes a deep learning model ResSwinT based on 2.5D multimodal MRI for the automatic classification of pediatric posterior fossa tumor (PA, MB, EP). Experimental results show that ResSwinT outperforms various classical and advanced deep learning models in multiple metrics such as classification accuracy and AUC, especially showing excellent performance in the classification tasks of PA and MB. By introducing a cross-attention mechanism, the model effectively fuses the local traits of Residual Network and the global traits of Swin Transformer, significantly improving the ability to identify subtle differences between different tumor types. Although there are still certain challenges in the EP classification task, this study verifies the feasibility and potential of the 2.5D strategy and MRI multi-sequence fusion. Meanwhile, through SHAP analysis, this study preliminarily revealed the contribution of key traits in the model decision-making process, further enhancing the interpretability of the model.

## Data Availability

The datasets presented in this article are not readily available because The datasets generated and/or analyzed during the current study are not publicly available due to the ongoing further studies but are available from the corresponding author on reasonable request. Requests to access the datasets should be directed to dongjie@ncwu.edu.cn.
